# Calcium desensitisation in late polymicrobial sepsis is associated with loss of vasopressor sensitivity in a murine model

**DOI:** 10.1186/s40635-014-0036-8

**Published:** 2015-01-31

**Authors:** Benjamin AJ Reddi, John F Beltrame, Richard L Young, David P Wilson

**Affiliations:** Intensive Care Unit, Royal Adelaide Hospital, North Terrace, Adelaide, South Australia 5000 Australia; Molecular Physiology of Vascular Function Laboratory, School of Medical Sciences, University of Adelaide, Frome Road, Adelaide, South Australia 5000 Australia; Discipline of Medicine, University of Adelaide, Frome Road, Adelaide, South Australia 5000 Australia; Basil Hetzel Institute, Queen Elizabeth Hospital, Woodville Road, Woodville, South Australia 5011 Australia

**Keywords:** Vascular tone, Sensitisation, Noradrenaline, Shock, Rho kinase, Myosin light-chain phosphatase

## Abstract

**Background:**

Sepsis is characterised by diminished vasopressor responsiveness. Vasoconstriction depends upon a balance: Ca^2+^-dependent myosin light-chain kinase promotes and Ca^2+^-independent myosin light-chain phosphatase (MLCP) opposes vascular smooth muscle contraction. The enzyme Rho kinase (ROK) inhibits MLCP, favouring vasoconstriction. We tested the hypothesis that ROK-dependent MLCP inhibition was attenuated in late sepsis and associated with reduced contractile responses to certain vasopressor agents.

**Methods:**

This is a prospective, controlled animal study. Sixteen-week-old C57/BL6 mice received laparotomy or laparotomy with caecal ligation and puncture (CLP). Antibiotics, fluids and analgesia were provided before sacrifice on day 5. Vasoconstriction of the femoral arteries to a range of stimuli was assessed using myography: (i) depolarisation with 87 mM K^+^ assessed voltage-gated Ca^2+^ channels (L-type, Ca_v_1.2 Ca^2+^ channels (LTCC)), (ii) thromboxane A_2_ receptor activation assessed the activation state of the LTCC and ROK/MLCP axis, (iii) direct PKC activation (phorbol-dibutyrate (PDBu), 5 μM) assessed the PKC/CPI-17 axis independent of Ca^2+^ entry and (iv) α_1_-adrenoceptor stimulation with phenylephrine (10^−8^ to 10^−4^ M) and noradrenaline (10^−8^ to 10^−4^ M) assessed the sum of these pathways plus the role of the sarcoplasmic reticulum (SR). ROK-dependent MLCP activity was indexed by Western blot analysis of P[Thr855]MYPT. Parametric and non-parametric data were analysed using unpaired Student's *t*-tests and Mann-Whitney tests, respectively.

**Results:**

ROK-dependent inhibition of MLCP activity was attenuated in both unstimulated (*n* = 6 to 7) and stimulated (*n* = 8 to 12) vessels from mice that had undergone CLP (*p* < 0.05). Vessels from CLP mice demonstrated reduced vasoconstriction to K^+^, thromboxane A_2_ receptor activation and PKC activation (*n* = 8 to 13; *p* < 0.05). α_1_-adrenergic responses were unchanged (*n* = 7 to 12).

**Conclusions:**

In a murine model of sepsis, ROK-dependent inhibition of MLCP activity in vessels from septic mice was reduced. Responses to K^+^ depolarisation, thromboxane A_2_ receptor activation and PKC activation were diminished *in vitro* whilst α_1_-adrenergic responses remained intact. Inhibiting MLCP may present a novel therapeutic target to manage sepsis-induced vascular dysfunction.

## Background

Septic shock arises when infection causes direct tissue injury and a maladaptive host response culminating in refractory vasodilation and loss of vasopressor sensitivity. Clinically, this manifests as septic shock and multi-organ failure, which even in modern intensive care units heralds a 90-day mortality rate of over 30% [1,2].

**Figure 1:**
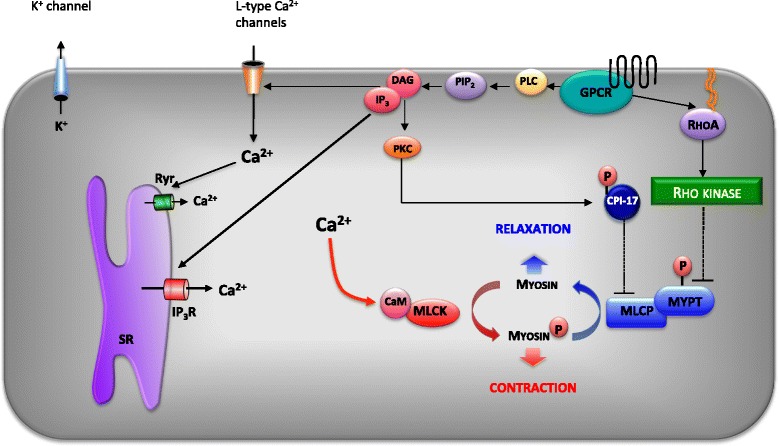
**Smooth muscle cell contraction depends on Ca** ^**2+**^ **-dependent and Ca** ^**2+**^ **-independent pathways.** Actin-myosin cross bridge cycling and smooth muscle contraction depend on phosphorylation of the myosin light chain by Ca^2+^: calmodulin (CaM)-dependent myosin light-chain kinase (MLCK). Relaxation is favoured by myosin light-chain phosphatase (MLCP) as the active subunit removes the phosphoryl group from the myosin light chain. Cytosolic [Ca^2+^] is derived from (i) the extracellular space (through voltage-operated Ca^2+^ channels (VOCC; predominantly Ca_v_1.2 L-type Ca^2+^ channels) and receptor-operated non-specific cation channels (ROCC)) or (ii) IP_3_-dependent release from the sarcoplasmic reticulum (SR). MLCP is inhibited, favouring contraction, either through (i) direct inhibition by PKC-dependent CPI-17 or (ii) inhibitory phosphorylation of the regulatory subunit MYPT by RhoA-dependent Rho kinase. α_1_-adrenergic G protein-coupled receptors (GPCR) activate all four contractile pathways: VOCC/ROCC, IP_3_/SR, PKC/CPI-17 and RhoA/ROK; TxA_2_ GPCR activate VOCC and RhoA/ROK; PDBu activates PKC/CPI-17 whilst high-[K^+^]-mediated depolarisation activates VOCC. PLC, phospholipase C; PIP_2_, phosphatidylinositol 4,5-bisphosphate; DAG, diacylglycerol; IP_3_, inositol triphosphate.

Vascular smooth muscle (VSM) contraction is determined by the balance of two opposing enzymatic processes: (i) myosin light-chain kinase (MLCK) phosphorylation of myosin enables actin-myosin cross bridge cycling and contraction; (ii) myosin light-chain phosphatase (MLCP) dephosphorylation of myosin uncouples actin-myosin, favouring relaxation. Activation of MLCK depends on cytosolic calcium (Ca^2+^) binding to calmodulin. Cystosolic Ca^2+^ is derived from either the sarcoplasmic reticulum (SR) or the extracellular space through, for example, voltage-gated L-type Ca_v_1.2 Ca^2+^ channels (LTCC). Vasoconstrictors variably modulate the cytosolic [Ca^2+^] and the degree of inhibition of MLCP to regulate vascular tone. MLCP can be inhibited either by (i) RhoA activation of Rho kinase (ROK), which inhibits MLCP by phosphorylating Thr855 of the MYPT regulatory subunit of MLCP [3-5], or (ii) PKC-mediated phosphorylation of Thr38 CPI-17, which directly inhibits MLCP [6-8]. Inhibiting MLCP promotes VSM contraction independent of the prevailing intracellular [Ca^2+^] - ‘Ca^2+^ sensitisation’. On the other hand, increasing MLCP activity by reducing Thr855 phosphorylation of MYPT or reduced Thr38 CPI-17 phosphorylation shifts the [Ca^2+^]:force relationship to the right - effectively reducing Ca^2+^ sensitivity. Individual vasopressors vasoconstrict through different signalling pathways; for instance, thromboxane is bimodal, activating LTCC and RhoA/ROK [3], whilst α_1_-adrenergic receptor agonists are multimodal, inducing vasoconstriction through two sources of Ca^2+^ (SR and LTCC) and two MLCP inhibitory pathways (RhoA/ROK and PKC/CPI-17) [9]. A figure summarising these contractile signalling pathways is provided (Figure 1).

There is evidence that Ca^2+^ desensitisation contributes to septic shock: in a rat model, acute endotoxaemia was associated with augmented MLCP activity and reduced sensitivity to an α_1_-adrenergic agonist [10]. Other models of sepsis have identified a rightward shift in the cytosolic [Ca^2+^]: force relationship [11] and attenuated vasopressor responses despite the presence of augmented cytosolic [Ca^2+^] [12], suggesting increased activity of MLCP. Many investigations into the vascular pathology of septic shock have focused on the early, hyperdynamic phase of sepsis. Importantly, however, 70% of deaths from sepsis occur after the first 3 days [2,13,14]. Mechanisms of vascular dysfunction at this later stage of disease remain relatively unexplored, but important differences have been identified; specifically, whilst early sepsis is associated with impaired vascular responses to α_1_-adrenergic agonists *in vitro* and a reduction in cytosolic Ca^2+^ entry, in the later phases of sepsis, the *in vitro* response to α_1_-adrenergic agonists and Ca^2+^ flux are restored [15,16], as is the *in vivo* blood pressure response to moderate doses of phenylephrine [17]. However, sensitivity to local vasoconstriction by thromboxane A_2_ (TxA_2_) and direct electrical coupling of the VSM membrane, significant for their importance in regulating regional blood flow [18], has not been evaluated in sepsis. Unlike α_1_-adrenergic agonists, TxA_2_ and membrane depolarisation do not stimulate SR Ca^2+^ release or PKC/CPI-17-mediated inhibition of MLCP and are therefore potentially more vulnerable to attenuation of the RhoA/ROK pathway [3,19]. Diminished efficacy of endogenous TxA_2_ and direct depolarisation secondary to dysfunction of the RhoA/ROK pathway may contribute to systemic hypotension in septic shock and dysregulation of regional blood flow [18,20].

To address this gap in knowledge, we used a murine caecal ligation and puncture (CLP) model of sepsis that included an intensive care treatment regimen that simulated the clinical experience of a septic patient [21,22]. CLP is a well-described, reproducible model of sepsis incorporating polymicrobial infection and tissue necrosis [23,24]. As an animal model, it replicates many of the physiological, haemodynamic and metabolic sequelae of sepsis [25-28]. We used a 5-day model of sepsis to identify mechanisms that contribute to vasopressor insensitivity during the late stages of sepsis, noting that most deaths from sepsis occur after the first 3 days [2,13,14]. We hypothesised that CLP sepsis would be associated with increased MLCP activity and Ca^2+^ desensitisation and that this activity would be associated with a reduction in *in vitro* vascular responses to vasoconstrictors such as thromboxane A_2_, phorbol ester and high K^+^ which are uni- or bimodal, engaging one or two signalling pathways. We hypothesised that responses to α_1_-adrenergic agonists which invoke multimodal mechanisms of Ca^2+^ entry (LTCC and SR Ca^2+^) and Ca^2+^ sensitisation (PKC/CPI-17 and Rho/ROK) would be relatively preserved *in vitro*.

## Methods

### Mice

All animal procedures were approved by the Animal Ethics Committees of the Institute of Medical and Veterinary Science (IMVS; Adelaide, South Australia) and the University of Adelaide (Adelaide, South Australia). Twenty-six, 16-week-old, male C57/BL6 mice purchased from the Animal Resources Centre, WA, were housed in the IMVS Animal Care Facility in individual cages following surgery with food and water *ad libitum*. Changes in clinical status were determined by cumulative clinical record scores as outlined previously [25].

### Caecal ligation and puncture

Sixteen mice underwent a midline laparotomy under general anaesthetic (1% to 1.5% isoflurane in oxygen) followed by location and ligation of the caecum 5 mm from the ileocaecal valve. A 21-gauge needle was used to puncture the caecum midway between the ligature and the tip of the caecum. Faecal material was expressed from the caecum into the abdominal cavity through the puncture site before the perforated caecum was placed back into the abdominal cavity and the incision was sutured. Morbidity associated with CLP surgery was assessed by monitoring for weight loss, abnormal temperatures (normal core temperature range 34 to 37) and other morbidity indicators including ruffled coat, hunched posture, reduced mobility, diarrhoea, abdominal distention, loss of righting ability and laboured respiration; these parameters were scored to calculate a cumulative disease index [25]. *Post*-*mortem* was carried out on representative CLP mice demonstrating acute suppurative and necrotising peritonitis. Ten mice underwent laparotomy without CLP as sham controls. All mice underwent an intensive care regime post-surgery: twice-daily subcutaneous administration of antibiotic (Baytril (enrofloxacin), 0.03 mg kg^−1^, 50 μl), fluid (saline 1 ml) for 5 days and analgesia (butorphanol, 0.05 mg kg^−1^, 25 μl) for 2 days. On day 5, surviving mice were killed with pentobarbitone (60 mg kg^−1^ i.p.) before tissue collection.

### Isolation of vessels

Femoral and caudal arteries were placed in cold Ca^2+^-free normal HEPES-Tyrode buffer containing, in mM, 135.5 NaCl, 5.9 KCl, 1.2 MgCl_2_, 11.6 glucose and 11.6 HEPES, pH 7.4, dissected free of adventitia and cut into 2-mm segments; femoral and caudal arteries were 250 and 300 μm in diameter, respectively. Caudal artery segments from sham and CLP treatment groups were snap-frozen in liquid nitrogen and stored at −80°C prior to SDS-PAGE/Western blot analysis.

### Vascular myography

Femoral arterial segments from sham and CLP treatment groups were mounted on a DMT 610M wire myograph (DMT, Aarhus, Denmark) to quantify isometric tension following stimulation. Following length tension analysis, femoral arteries were set at an optimal resting tension of 3 mN and equilibrated in normal HEPES-Tyrode buffer (in mM, 135.5 NaCl, 5.9 KCl, 1.2 MgCl_2_, 2.5 CaCl_2_, 11.6 glucose, 11.6 HEPES, pH 7.4). The arteries were equilibrated for 30 min before being stimulated three times with a depolarising stimulus of 87 mM K^+^-HEPES-Tyrode buffer (K^+^HT), in which NaCl was substituted with KCl to maintain an iso-osmolar solution. Following relaxation in NHT buffer, the arteries were incubated with the stable thromboxane A_2_ receptor agonist U46619 (0.1 μM), the direct PKC activator phorbol-dibutyrate (PDBu) (5 μM), the α_1_-adrenergic agonist phenylephrine (PE) (10^−8^ to 10^−4^ M) or noradrenaline (NA) (10^−8^ to 10^−4^ M), primarily an α_1_-adrenergic agonist but with significant β-adrenergic agonist action. Noradrenaline was used because, although less specific in action than phenylephrine, it is more commonly used both in sepsis research and clinical practice.

Following a 10-min stimulation with U46619 (0.1 μM) or PDBu (5 μM) or generation of a dose-response relationship with NA or PE (10^−8^ to 10^−4^ M), tissues were snap-frozen in dry ice-cold 10% trichloroacetic acid/acetone, washed with dry ice-cold acetone and stored at −80°C prior to SDS-PAGE/Western blot analysis.

Myography data and biochemical analysis of stimulated arterial tissue was derived from the femoral artery. As quantities of the femoral artery were limited, biochemical analysis of unstimulated sham and CLP tissue was derived from the caudal artery (nb caudal segments were always compared with caudal and femoral with femoral). Endothelial integrity of arterial segments was confirmed by phenylephrine challenge followed by an intact 90% ACh relaxation.

### Western blot analysis of MYPT

For analysis of total MYPT and P[Thr855]MYPT, proteins were extracted from each 2-mm arterial segment using 100 μl of sample buffer containing 50 mM Tris-HCl, pH 6.8, 1% (*w*/*v*) SDS, 1× Complete™ protease inhibitor cocktail (Roche, Mannheim, Germany), 100 μM di-isopropylfluorophosphate (Sigma-Aldrich, Caste Hill, Australia), 10 mM DTT, 10% (*w*/*v*) sucrose and 0.1% (*w*/*v*) bromophenol blue. Samples were heated to 95°C for 5 min and then agitated for 30 min prior to SDS-PAGE using a Bio-Rad (Sydney, Australia) Mini-PROTEAN II unit at 200 V for 60 min. For analysis of MYPT, proteins were transferred, using a Bio-Rad Mini transfer unit, onto 0.22 μm nitrocellulose (Bio-Rad) at 100 V for 30 min in transfer buffer containing 25 mM Tris-HCl, 192 mM glycine, 0.1% (*w*/*v*) SDS and 20% (*v*/*v*) methanol.

Following protein transfer to nitrocellulose, non-specific binding sites were blocked with 50% LI-COR Odyssey blocking buffer (LI-COR Biosciences, Lincoln, NE, USA) for 60 min, followed by incubation with TBS-T (20 mM Tris, 150 mM NaCl, 0.05% (*v*/*v*) Tween-20) containing either a mouse-derived affinity-purified anti-MYPT antibody (1:1,000) made in-house or a commercially available (Upstate Biotechnology, Lake Placid, NY, USA) rabbit-derived anti-P[Thr855]MYPT antibody (1:1,000) for 60 min. Nitrocellulose membranes were washed three times in TBS-T and incubated with a 1:10,000 dilution (in TBS-T) of biotin-conjugated secondary antibodies (Pierce Thermo Scientific, Rockford, IL, USA): anti-mouse IgG for MYPT and anti-rabbit IgG for P[Thr855]MYPT (1:10,000), for 60 min before another three washes with TBS-T. The nitrocellulose was then incubated for 60 min in TBS-T with a 1:10,000 dilution of streptavidin conjugated to the 800-nm DyLight fluorochome (Pierce Thermo Scientific); fluorescence was detected and quantified using the LI-COR Odyssey system. Western blot signals of the Thr855 phosphorylation state of MYPT of untreated vs. U46619 (0.1 μM)- or PDBu (5 μM)-treated rat caudal artery as previously published [3] were used as controls.

Although consistency of protein content was confirmed with Coomassie blue-stained actin, all Western blot signals were evaluated and found to be within the linear range of detection. Vessel size and protein load were not significantly different; nevertheless, the primary outcome of phosphorylation analysis is expressed as a ratio of P[Thr855]MYPT to total MYPT taken from the same sample.

### Statistical analysis

The Mann-Whitney test was used for non-parametric data comparisons, and Student's *t*-test was used to compare parametric data (both two-tailed); *p* < 0.05 was considered statistically significant. Asterisks indicate statistically significant differences from control; data are presented as mean ± SEM.

## Results

### Morbidity and mortality

There were no deaths in sham-operated mice. The mortality rate in CLP-operated mice was 16%, similar to the real-world experience of severe sepsis managed in a critical care environment [29,30]. The cumulative disease index was significantly higher for mice undergoing CLP surgery than for sham-operated controls (*p* < 0.05, data not shown) [25].

### Does the ROK-dependent basal activity state of myosin phosphatase, indexed by Thr855 MYPT phosphorylation, differ between septic and control mice?

**Figure 2:**
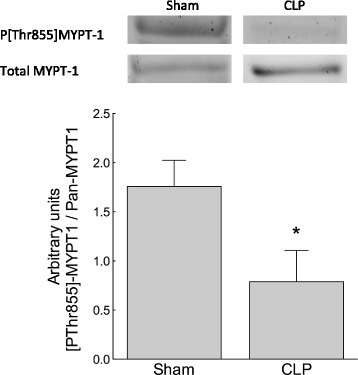
**Sepsis was associated with increased activation state of MLCP.** The activity state of MLCP was greater in isolated, unstimulated caudal arteries from 5-day CLP mice than in those from sham mice. The increased activity state of MLCP was indexed by reduction in the ratio of Thr855 phosphorylated to unphosphorylated MYPT determined by Western blot analysis of snap-frozen vessel segments. *p < 0.05, *n* = 6 to 7.

To compare the basal activity state of MLCP in sham and CLP mice, the proportion of total MYPT in the [Thr855]-phosphorylated, i.e. ROK-inhibited, state was identified using Western blot analysis of unstimulated mouse caudal artery segments. The proportion of [Thr855]-phosphorylated MYPT in unstimulated vessels was greater in sham than in CLP mice (*n* = 6 to 7; *p* < 0.05), indicating that MLCP was more active in CLP mice favouring vasorelaxation (Figure 2).

### Does the ability of thromboxane A_2_ receptor stimulation to inhibit MLCP differ in CLP and sham mice?

**Figure 3:**
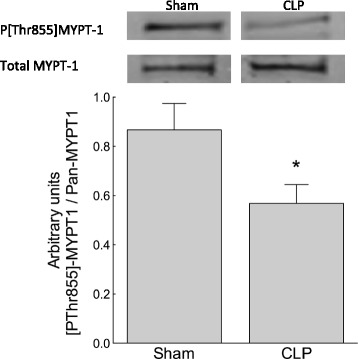
**TxA**_**2**_ **receptor-mediated inhibition of MLCP was attenuated in sepsis.** The activity state of MLCP was increased in femoral arteries stimulated with U46619 (0.1 μM) in 5-day CLP mice relative to those in sham mice (indexed by reduction in the ratio of Thr855 phosphorylated to unphosphorylated MYPT on Western blot analysis). *p < 0.05, *n* = 9 to 12.

Thromboxane activates the RhoA/ROK pathway leading to inhibitory phosphorylation of Thr855 MYPT, the regulatory subunit of MLCP, favouring contraction by Ca^2+^ sensitisation [3]. To establish whether the ability of the TxA_2_ receptor to inhibit MLCP was attenuated in CLP compared to sham mice, the [Thr855] phosphorylation state of MYPT was compared in isolated femoral artery segments following challenge with the TxA_2_ receptor agonist U46619 (0.1 μM). CLP mice showed reduced phosphorylation of [Thr855]MYPT (less MLCP inhibition) in response to TxA_2_ receptor stimulation than sham-treated mice (Figure 3; *n* = 9 to 12; *p* < 0.05).

### Was the reduction in Thr855 MLCP phosphorylation in CLP mice associated with a reduction in vascular contractility?

**Figure 4:**
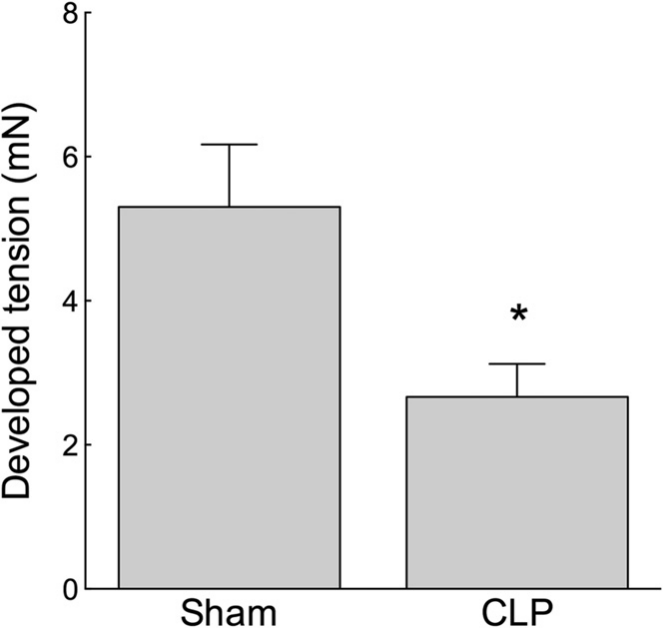
**The vascular contractile response to the thromboxane receptor agonist U46619 was attenuated in sepsis.** Wire myograph-mounted femoral arteries from 5-day CLP mice developed lower tension under stimulation with U46619 (0.1 μM) compared to those from sham mice. *p < 0.05, *n* = 10 to 12.

TxA_2_ elicits VSM contraction both through Rho kinase-mediated MLCP inhibition and by increasing the open probability of LTCC. If RhoA/ROK activity was disrupted during sepsis, decreasing [Thr855]MYPT phosphorylation, it would be expected that activation of TxA_2_ receptors would fail to fully restore MLCP inhibition. The consequence would be reduced Ca^2+^ sensitivity and a diminished contractile response to the TxA_2_ mimetic U46619. The contraction of isolated femoral artery segments in response to U46619 (0.1 μM) was attenuated in CLP compared to sham mice (Figure 4; *n* = 10 to 12; *p* < 0.05), consistent with the hypothesis that reduced MLCP inhibition contributes to septic shock.

### Was the contractile response to PDBu, a direct activator of PKC/CPI-17, attenuated in sepsis?

**Figure 5:**
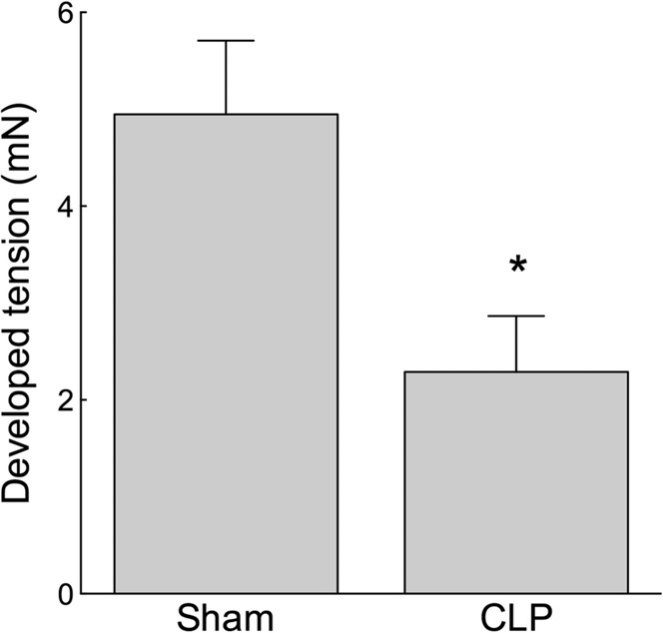
**The vascular contractile response to the direct PKC activator phorbol-dibutyrate (PDBu) was attenuated in sepsis.** Wire myograph-mounted femoral arteries from 5-day CLP mice developed lower tension under stimulation with PDBu (5 μM) compared to those from sham mice. *p < 0.05, *n* = 8 to 13.

**Figure 6:**
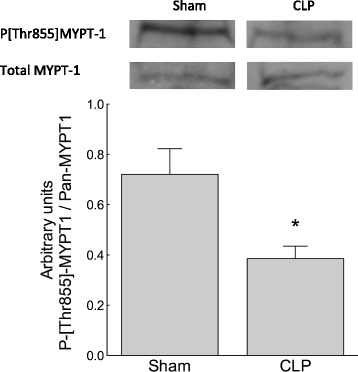
**Attenuated PKC-dependent contraction in septic mouse artery was associated with reduced inhibitory phosphorylation of MLCP.** The activity state of MLCP was increased in femoral arteries stimulated with PDBu (5 μM) in 5-day CLP mice relative to those in sham mice (indexed by reduction in the ratio of Thr855 phosphorylated to unphosphorylated MYPT on Western blot analysis). *p < 0.05, *n* = 8.

The direct PKC agonist PDBu inhibits MLCP by stimulating PKC-dependent phosphorylation of Thr38 of CPI-17 [31]. In the Thr38-phosphorylated state, CPI-17 is a specific, direct inhibitor of MLCP [6]. Contractile responses to PDBu (5 μM) were reduced in femoral artery segments from CLP mice compared with segments from sham mice (Figure 5; *n* = 8; *p* < 0.05). As expected, treatment of artery segments from control mice with PDBu did not directly alter the Thr855 phosphorylation of MYPT (data not shown) [19]. To identify whether the attenuated contractile response to PDBu in CLP mice may have resulted from a sepsis-dependent reduction in baseline Thr855 phosphorylation of MYPT and consequently augmented MLCP activity, we used Western blot analysis to ascertain the Thr855 phosphorylation state of MYPT in control and CLP mice. We identified a reduction in Thr855 phosphorylation of MYPT in the PDBu (5 μM)-treated femoral artery segments from CLP mice compared to segments from sham mice (Figure 6; *n* = 8; *p* < 0.05).

### Could the attenuated contractile response to TxA_2_ receptor and PKC/CPI-17 stimulation in CLP mice be wholly explained by changes in membrane potential during sepsis?

**Figure 7:**
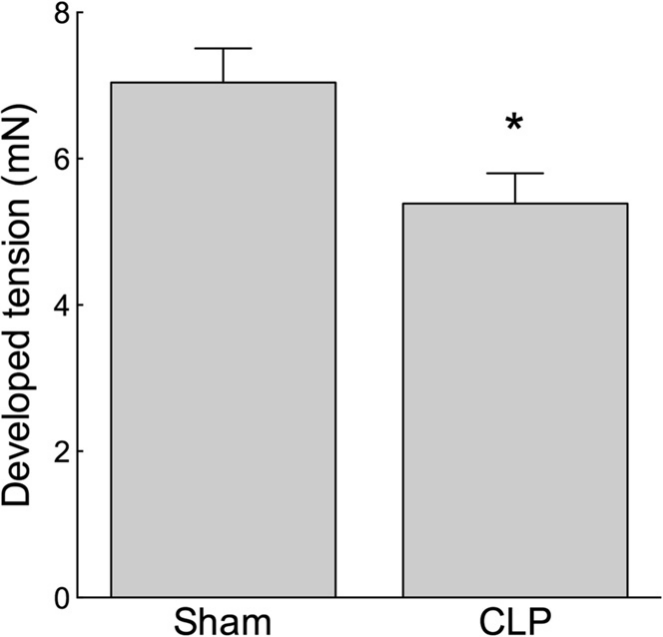
**The vascular contractile response to depolarisation with high-K** ^**+**^ **HT solution was attenuated in sepsis.** Wire myograph-mounted femoral arteries from 5-day CLP mice developed lower tension under stimulation with a depolarising high-K^+^ HT solution compared to those from sham mice. *p < 0.05, *n* = 9 to 13.

A significant body of work has demonstrated that augmented ATP-sensitive potassium (K_ATP_) channel function causes VSM hyperpolarisation with consequent vasodilation and vasopressor resistance in sepsis [32,33]. Contractile responses to U46619 and PKC/CPI-17 depend on both the basal activity of MLCP and baseline conductance of the K_ATP_ channel as it modulates membrane potential and hence the activity of LTCC. In order to isolate the contributions made by increased MLCP and K_ATP_ channel activities to attenuated functional vascular contractile responses, we made use of a high-K^+^ depolarising solution, which directly activates LTCC independent of K_ATP_ channel conductance. The contractile response to high-K^+^-mediated depolarisation of isolated femoral artery segments from CLP mice was reduced compared to those from sham mice independent of K_ATP_ activity (Figure 7; *n* = 9 to 13; *p* < 0.05).

### Is the contractile response to α_1_-adrenergic stimulation intact during the later phase of sepsis?

**Figure 8:**
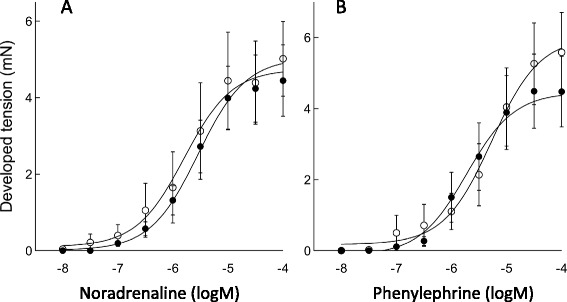
**Vasoconstrictor responses to stimulation with phenylephrine and noradrenaline remained intact in arteries from septic mice.** Dose-response curves to stimulation with NA (10^−8^ to 10^−4^ M) **(A)** and PE (10^−8^ to 10^−4^ M) **(B)** were generated using wire myograph-mounted femoral arteries. For both agonists, dose-response curves in 5-day CLP mice (filled circles) and sham mice (hollow circles) did not differ with regard to EC_50_ or *E*_max_ (*p* > 0.05). (*n* = 7 to 12).

VSM stimulation by α_1_-adrenergic agonists involves Ca^2+^ entry through LTCC and from SR as well as Ca^2+^ sensitisation mediated by both PKC/CPI-17 and RhoA/ROK activation [9]. Contrary to acute endotoxaemia models, studies analysing vessels after longer periods of sepsis have found recovery of contractile responses to α_1_-adrenergic stimulation [16]. We used isolated femoral vessels from a 5-day model of polymicrobial sepsis, somewhat akin to a clinical scenario, to identify whether survivors had recovered α_1_-adrenergic response. Figure 8 shows that the contractile response to the α_1_-adrenergic agonists noradrenaline (Figure 8A) and phenylephrine (Figure 8B) (10^−8^ to 10^−4^ M) was not significantly different in CLP compared to sham mice (*E*_max_ and EC_50_*p* = NS, *n* = 7 to 12).

## Discussion

Hospital mortality from septic shock remains around 30%, highlighting the need for a more complete understanding of the mechanisms of pathological vasodilation [1]. Proposed mechanisms include reduced α_1_-receptor density and reduced α_1_-adrenergic signal transduction [34], activation of vascular K_ATP_ channels [33], impaired SR Ca^2+^ release [15,35], relative corticosteroid and vasopressin deficiency [36,37] and activation of inducible nitric oxide synthase [38] and vasodilating inflammatory mediators [39-42]. We tested the hypothesis that reduced inhibition of MLCP promotes Ca^2+^ desensitisation, contributing to pathological vasodilation in a murine CLP model of sepsis. Normally, MLCP inhibition sensitises the vascular contractile apparatus to Ca^2+^, favouring vasoconstriction [19,43]. Events that release MLCP inhibition by reducing either (i) RhoA/ROK-mediated inhibitory Thr855 phosphorylation of the regulatory subunit MYPT or (ii) PKC-mediated Thr38 phosphorylation of CPI-17 favour vasorelaxation [19]. Using a 5-day murine model of polymicrobial sepsis, we identified that both the baseline phosphorylation state of Thr855 MYPT and the Thr855 phosphorylation state of MYPT following stimulation with the TxA_2_ receptor agonist U46619 (0.1 μM) were attenuated in septic animals compared to sham-operated controls (Figures 2 and 3). Reduced Thr855 MYPT phosphorylation was associated with reduced vasoconstrictor response to U46619, PDBu or high-[K^+^]-mediated depolarisation (Figures 4, 5 and 7). These data are consistent with late sepsis being associated with Ca^2+^ desensitisation, rendering the vasculature less responsive to certain endogenous vasopressors.

Previous data are consistent with the view that vasopressor responses can be attenuated in sepsis due to Ca^2+^ desensitisation, rather than as a consequence of reduced flux of Ca^2+^ into the cytosol [Ca^2+^]_cyt_. For example, the [Ca^2+^]_cyt_ content in the aortae of rats with confirmed CLP sepsis was actually elevated relative to that of non-septic animals [12]. Isolated mesenteric arteries from rats injected with *Escherichia coli* lipopolysaccharide (LPS) also had elevated VSM [Ca^2+^]_cyt_ and a force:intracellular Ca^2+^ relationship shifted to the right, consistent with Ca^2+^ desensitisation [11]. More recently, in an acute (6 to 24 h) rat model of LPS-induced endotoxaemia, da Silva-Santos and colleagues identified reduced inhibitory phosphorylation of MLCP in the mesenteric vasculature, associated with reduced vasopressor sensitivity [10].

Sepsis in humans typically lasts longer than most experimental animal models of sepsis, which, for practical reasons, are often less than 24 h. In clinical practice, most deaths occur after several days of sepsis [2,13,14]. It is arguable that a CLP model, whereby tissue necrosis and polymicrobial infection with host enteric organisms develop over several days after traumatic perforation of a viscus, reflects a more common clinical paradigm than sepsis induced by injection of bacterial inoculum or LPS [44,45]. We evaluated the functional and molecular features of murine vascular dysfunction 5-days post caecal ligation and puncture.

Our data identified an important difference in vascular response between acute endotoxaemia and a 5-day peritonitis model: the first 2 to 6 h of acute endotoxaemia models typically demonstrate reduced vasoconstriction in response to α_1_-adrenoceptor stimulation [10,16,33,46]. In contrast, after 5 days of polymicrobial sepsis, we identified normal *in vitro* α_1_-adrenoceptor-mediated vasoconstriction, consistent with endotoxaemia models, which found recovery of *in vitro* and *in vivo* sensitivity to moderate doses of α_1_-adrenoceptor agonist between 6 and 24 h [16,17]. However, our data identifies clear insensitivity to other vasoconstrictor mechanisms, including TxA_2_ receptor activation and direct membrane depolarisation (Figures 4, 5 and 7). α_1_-adrenoceptor agonist responses may demonstrate resilience by recruiting multiple, parallel signalling pathways to achieve contraction (Figure 1), specifically 1) LTCC opening, 2) SR Ca^2+^ release, 3) inhibition of MLCP by PKC/CPI-17 and 4) inhibition of MLCP by RhoA/ROK phosphorylation of MYPT. In particular, α_1_-adrenoceptor agonists increase cytosolic [Ca^2+^]_cyt_ through SR Ca^2+^ release, a mechanism not shared by TxA_2_ receptor, PDBu activation or high-K^+^-mediated contraction [3,19], and have two mechanisms by which to inhibit MLCP through both PKC/CPI-17 and RhoA/ROK. These data provide a mechanistic explanation for the effectiveness of α_1_-adrenoceptor agonists as vasoconstrictors in sepsis.

The results of our study together with those of da Silva-Santos and colleagues [10] indicate two salient findings: 1) disinhibition of MLCP favouring Ca^2+^ desensitisation has been identified at different time points and in different models of sepsis, suggesting a potentially important role in the pathogenesis of septic shock, and 2) preservation of *in vitro* α_1_-adrenoceptor agonist sensitivity may not necessarily reflect intact vasoconstrictor responses to other physiologically significant stimuli.

Several potential mechanisms by which sepsis might promote MLCP disinhibition and Ca^2+^ desensitisation can be postulated. Pathogenic bacteria produce toxins capable of directly interfering with RhoA/ROK-dependent phosphorylation of MYPT, thereby disinhibiting MLCP and opposing cell contraction [19]: *E. coli* and *Clostridium botulinum* derived exotoxins EDIN and C3-transferase ADP-ribosylate and inactivate RhoA, *Clostridium difficile* toxin B glycosylates and inactivates RhoA whilst *Yersinia* spp. produces toxin Yop T which prevents RhoA from co-localising in the cell membrane with Rho kinase [47-49]. In contrast, SpA from *Staphylococcus aureus* and CNF1 from *E. coli* promote RhoA/ROK activity [50,51]. Endogenous mediators might also contribute to Ca^2+^ desensitisation: nitric oxide, kynurenine and ANP levels are elevated in septic shock [40,52], potentially promoting protein kinase G (PKG)-dependent disinhibition of MLCP and Ca^2+^ desensitisation [10,53,54].

Our findings of impaired response to TxA_2_ and membrane depolarisation may be of particular clinical significance for two reasons: Firstly, it is well established that restoration of normal systemic circulatory parameters is not necessarily indicative of restored regional perfusion, and indeed, ongoing regional ischaemia despite restoration of systemic blood pressure is associated with poor clinical outcome [55-58]. Microvascular splanchnic perfusion is not only reduced but highly heterogeneous in septic shock even within areas of uniform metabolic demand [20], indicating a failure of local vasomotor regulation. Since TxA_2_ [59] and local VSM membrane potential [60,61] play a key role in local vasomotor regulation, our evidence that their effectiveness is impaired in late sepsis and might be restored by MLCP inhibition opens a potentially useful area of investigation. Secondly, the experimentally observed restoration of α_1_-adrenergic responses *in vitro* may not be borne out in the septic patient because *in vivo* endogenous vasoconstrictors such as constitutively secreted TxA_2_ and direct depolarisation potentiate the response to an α_1_-adrenergic agonist [62]. The apparently attenuated response to α_1_-adrenergic agonists *in vivo* may reflect diminished response to these normally synergistic vasopressor pathways.

### Implications for clinical research and management

Our data, from a murine model, identify the association between MLCP disinhibition and vasopressor insensitivity in a murine model of sepsis. Future studies relating MLCP activity and regional blood flow *in vivo* in large animals and ultimately human sepsis patients will provide valuable insights into the specific clinical consequences of myosin phosphatase dysregulation.

Advances in our understanding of the pathobiology of sepsis have characterised sepsis as a heterogeneous disease, and optimal therapy will need to be tailored to particular unique patient/pathogen disease phenotypes [63,64]. Our findings suggest that in certain patients, excessive MLCP activity could contribute to the pathogenesis of sepsis. Identifying these patients would allow targeted therapeutic inhibition of MLCP or use of vasopressors less dependent upon RhoA/ROK signalling. Restoring vascular tone by directly inhibiting MLCP circumvents receptor downregulation and avoids promoting injurious increases in cytosolic [Ca^2+^] [12]. In addition, targeting MLCP-dependent Ca^2+^ sensitisation could be particularly effective in re-establishing local responsiveness to TxA_2_ and membrane depolarisation, thereby restoring regulation of regional perfusion [18].

The role of RhoA/ROK-mediated inhibition of MLCP extends beyond vascular smooth muscle: thrombin, a key mediator of the cross-talk between coagulation and inflammation in sepsis has, been shown to inactivate MLCP and therefore contraction in a RhoA-dependent manner in human endothelial cells [65]. Given the prominence of endothelial dysfunction in sepsis, investigating the role of MLCP disinhibition in the pathogenesis of increased endothelial permeability is a research priority.

### Study limitations

We studied an animal model of sepsis at a single time point, and translating our results to patients mandates caution. To reflect human sepsis as closely as possible, we used a CLP model, which has advantages over bacterial inoculum and lipopolysaccharide models [45], incorporating fluid and antibiotic therapy to recreate haemodynamic and metabolic phases of treated human polymicrobial sepsis. Mortality in the intervention arm was 16%, comparable to mortality in human studies of severe sepsis, but approximately half the mortality rate found amongst patients with septic shock [30]. In addition, to maintain consistency, we analysed vessels from sacrificed mice rather than mice dying of their sepsis. For these reasons, our data may be derived from somewhat healthier animals than typical patients with septic shock.

## Conclusions

Mechanisms of pathological vasodilation and vasopressor resistance in septic shock are diverse. Our observations in a murine caecal ligation and puncture model are consistent with the hypothesis that during sepsis disinhibition of myosin phosphatase promotes insensitivity to certain physiologically important vasoconstrictor mechanisms, such as thromboxane A_2_ and membrane depolarisation. Approaches aimed at directly inhibiting MLCP might prove to be an effective therapeutic strategy to counter the pathological vasodilatation of septic shock.
